# Texture analysis in ^177^Lu SPECT phantom images: Statistical assessment of uniformity requirements using texture features

**DOI:** 10.1371/journal.pone.0218814

**Published:** 2019-07-31

**Authors:** Anna Sarnelli, Emilio Mezzenga, Alessandro Vagheggini, Filippo Piccinini, Giacomo Feliciani, Maria Luisa Belli, Francesco Monti, Marta Cremonesi, Corrado Cittanti, Giovanni Martinelli, Giovanni Paganelli

**Affiliations:** 1 Medical Physics Unit, Istituto Scientifico Romagnolo per lo Studio e la Cura dei Tumori (IRST) IRCCS, Meldola, Forli-Cesena, Italy; 2 Unit of Biostatistics and Clinical Trials, Istituto Scientifico Romagnolo per lo Studio e la Cura dei Tumori (IRST) IRCCS, Meldola, Forli-Cesena, Italy; 3 Scientific Directorate, Istituto Scientifico Romagnolo per lo Studio e la Cura dei Tumori (IRST) IRCCS, Meldola, Forli-Cesena, Italy; 4 Istituto Europeo di Oncologia, Via Ripamonti, Milano, Italy; 5 Morphology, Surgery and Experimental Medicine Department, University of Ferrara, Ferrara, Italy; 6 Nuclear Medicine Unit, Istituto Scientifico Romagnolo per lo Studio e la Cura dei Tumori (IRST) IRCCS, Meldola, Forli-Cesena, Italy; University of Pennsylvania, UNITED STATES

## Abstract

The purpose of this study was to apply texture analysis (TA) to evaluate the uniformity of SPECT images reconstructed with the 3D Ordered Subsets Expectation Maximization (3D-OSEM) algorithm. For this purpose, a cylindrical homogeneous phantom filled with ^177^Lu was used and a total of 24 spherical volumes of interest (VOIs) were considered inside the phantom. The location of the VOIs was chosen in order to define two different configurations, *i*.*e*. gravity and radial configuration. The former configuration was used to investigate the uniformity of distribution of ^177^Lu inside the phantom, while the latter configuration was used to investigate the lack of uniformity from center towards edge of the images. For each VOI, the trend of different texture features considered as a function of 3D-OSEM updates was investigated in order to evaluate the influence of reconstruction parameters. TA was performed using CGITA software. The equality of the average texture feature trends in both spatial configurations was assumed as the null hypothesis and was tested by functional analysis of variance (fANOVA). With regard to the gravity configuration, no texture feature rejected the null hypothesis when the number of subsets increased. For the radial configuration, the statistical analysis revealed that, depending on the 3D-OSEM parameters used, a few texture features were capable of detecting the non-uniformity of ^177^Lu distribution inside the phantom moving from the center of the image towards its edge. Finally, cross-correlation coefficients were calculated to better identify the features that could play an important role in assessing quality assurance procedures performed on SPECT systems.

## Introduction

Today different imaging modalities are available to assist clinicians in their daily work by providing precious details for the detection and diagnosis of human diseases. Constant improvements in image acquisition devices have led to an increase in the diagnostic information within a single study and to the possibility of extracting “quantitative” features from both morphological and functional tomographic images. Consequently, new tools based not only on visual analysis but also on automated image analysis are available to support clinical decision making [1]. In 1979 the concept of image texture analysis (TA) was introduced to characterize the spatial relationship between gray levels in neighbouring pixels within a region of interest (ROI) in an image [2]. However, it took almost three decades to see the first pioneering publication investigating the use of TA in medical images [3]. Since then, many other studies have been carried out based on the use of TA methods under different clinical conditions and using non-invasive medical imaging modalities [4].

The discipline that studies and analyzes the features extracted from medical images is called *radiomics* [5] and its aim is to generate a predictive model combining the information provided by the TA with that of patient outcome to identify a specific clinical treatment. In particular, numerous studies have highlighted the potential of *radiomics* as a powerful tool to quantify the characteristics of tumors in a non-invasive manner [6], indicating its usefulness in both prognostic and predictive models. Typically, the workflow used in the application of the TA methods in medical images consists of a series of consecutive steps: 1) acquisition, reconstruction and selection of the images; 2) identification and segmentation of regions (ROIs) and volumes of interest (VOIs); 3) extraction of descriptive texture features from ROIs or VOIs; 4) feature selection and statistical correlation with clinical outcome; and 5) data classification. Another important step that should be included for the generation of a radiomics predictive model is external validation, an essential element for comparing the usefulness of the generated model between different research institutions/hospitals. In fact, different machines provide medical images with completely different characteristics [7], and this variability impairs the results of a predictive model and their generalization. One way to resolve this problem is to identify features that are stable during the analyses and sufficiently robust with respect to system characteristics and their acquisition/reconstruction parameters [8].

Although many studies have been published on the usefulness of TA methods in a clinical context [9–12], only a few deal with the use of TA methods for the quantitative evaluation of SPECT images [13]. TA applied to SPECT images could play an important role in diseases and treatments that utilize SPECT imaging, not only for diagnosis but also for patient-specific dosimetry purposes.

The SPECT images are affected by a “blobby” noise [14], which is strongly dependent on the algorithm and the parameters used in the SPECT image reconstruction process. One of the main parameters influencing the texture noise of a reconstructed image is the collimator-detector response (CDR), for which analytic and iterative reconstruction approaches have been implemented to compensate the CDR function. However, due to the oscillation of the signal in the image that jumps at sharp edges, resulting in an overshoot near them, CDR compensation can introduce artifacts typically known as Gibbs rings [15]. First-order statistics based on standard descriptors [16] cannot completely characterize the images in terms of texture and pattern because they do not consider the spatial relationship and correlation with neighboring pixel values. Recently, TA has been proven to be suitable for quantitative assessment of quality assurance procedures performed on SPECT systems where only qualitative statements are needed [13].

The present study evaluated SPECT system reconstruction performances by using TA on reconstructed ^177^Lu SPECT images of a homogeneous phantom, with the goal of finding potential metrics to test on different SPECT systems. This in order to define a different approach to SPECT image analysis that can use TA as a figure of merit to describe uniformity in SPECT images. We thus extracted a large number of texture features from VOIs defined on reconstructed SPECT images of the homogeneous phantom, analyzing the trend of several features as a function of the number of 3D-OSEM updates (*i*.*e*. subsets (S) and iterations (I)), considering as starting point the results of a previous study [17]. Finally, we statistically investigated possible correlations between different extracted features as a function of the reconstruction parameters and VOI location.

In brief, we: 1) statistically investigated the feature trends as a function of the reconstruction parameters to identify potential differences in spatial configuration of VOIs inside the SPECT phantom images; 2) studied the correlation between the features stable with respect to reconstruction parameters in order to better define the relationship between the texture features presenting significant differences; 3) explored the potential role of the TA as a new approach to the quantitative characterization of ^177^Lu SPECT images that is not fully achieved by a first-order statistics. Furthermore, to the best of our knowledge, the adoption of a functional analysis statistical approach, which allows us to consider the values of the features as a function of the reconstruction parameters, represents a novelty in TA applied to SPECT imaging.

## Materials and methods

**Fig 1:**
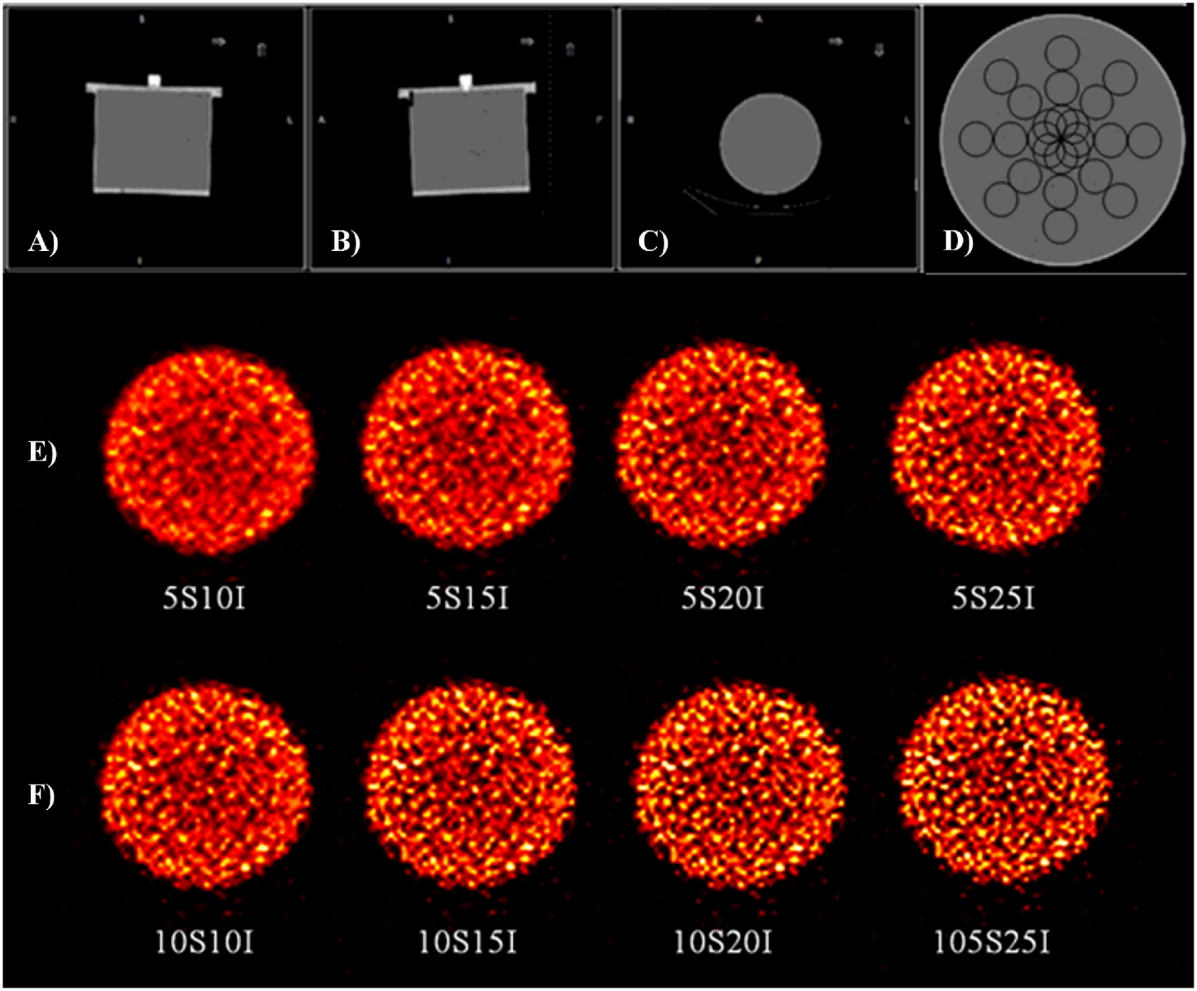
SPECT/CT images of the homogeneous cylindrical phantom. The top row shows CT images of the homogeneous phantom in A) coronal, B) sagittal and C) transaxial sections, together with D) VOI location used for texture analysis. The second and third rows show reconstructed SPECT images of the central slice of the phantom for E) 5S and F) 10S, and different I values.

SPECT acquisition related to a homogeneous cylindrical phantom filled with 0.11 MBq/mL of ^177^Lu was used to perform TA (Fig 1) [17]. In particular, SPECT acquisition was performed using a hybrid SPECT/CT system (Discovery NM/CT 670, GE Healthcare, Milwaukee, USA), equipped with two gamma detector heads (9.5 mm NaI(Tl) crystal thickness with 40 cm axial by 54 cm diameter field of view), and an integrated 16-slice-CT component (model: Bright Speed 16, GE Healthcare, Milwaukee, USA). SPECT projection data were acquired in three energy windows using a parallel-hole medium energy general purpose (MEGP) collimator: a symmetrical 20% wide energy window was centered at 208 keV ^177^Lu photopeak (energy window: 187.2 keV– 228.8 keV), together with two 8.7% and 11.8% wide adjacent scatter windows, representing the upper and lower scatter window, respectively. Acquisition was performed using 120 projections, non-circular step-and-shoot orbit, 128x128 matrix and 4.42x4.42 mm^2^ pixel size. The subsequent CT scan was acquired at 120 kV, 80 mAs, 1.375 pitch, 16x1.25 mm collimation and 3.75 slice thickness.

The SPECT was reconstructed on a dedicated workstation (Xeleris 3.1108, GE Healthcare, Milwaukee, USA) using a 3D-OSEM algorithm, including CDR, scatter correction and attenuation correction. The OSEM parameters used were 5S and 10S, while the number of I ranges from 10 to 25 in steps of 5, accordingly to the results reported in [17]. No pre- or post- reconstruction filter was used. In this way a total of 8 reconstructed datasets were obtained. On each dataset, 24 spherical VOIs of 19 cm^3^ in volume were contoured, and their location inside the phantom is shown in Fig 1D. With the aim to perform an analysis similar to that proposed in [18], the external VOIs were located at a distance of 15 mm between their surface and the inner edge of the phantom. This configuration was repeated twice in the inner portion of the phantom, with increasing overlapping of the VOIs.

CGITA software was used for TA [19]. In the analysis performed, a total of 69 texture features were computed and grouped as follows: 6 belong to Co-occurrence (C), 11 to Voxel Alignment (VA), 5 to Neighborhood Intensity Difference (NID), 11 to Intensity Size Zone (ISZ), 7 to Normalized Co-occurrence (NC), 11 to Voxel Statistics (VS), 2 to Texture Spectrum (TS), 3 to Texture Feature Coding (TFC), 8 to Texture Feature Coding Co-occurrence (TFCC) and 5 to Neighborhood Gray-Level Dependence (NGLD) matrix. The features used in this study are reported in Table 1. A detailed feature description is reported in [19] and references therein. Moreover, the analysis performed on the considered VOIs used the following CGITA settings: 1) local normalization performed with minimum and maximum values calculated inside the VOI; 2) the distance between pairs of voxels was set to one (direct neighbours); 3) the angular orientation is averaged and 4) digitization bins of 64.

**Table 1:** Texture features analized with CGITA software.

Parent matrix	Feature measure
Co-occurrence (C)	second angular moment, contrast, entropy, homogeneity, dissimilarity, inverse difference moment
Voxel Alignment (VA)	short-run emphasis, long-run emphasis, intensity variability, run-length variability, run percentage, low-intensity run emphasis, high-intensity run emphasis, low-intensity short-run emphasis, high-intensity short-run emphasis, low-intensity long-run emphasis, high-intensity long-run emphasis
Neighborhood Intensity Difference (NID)	coarseness, contrast, busyness, complexity, strength
Intensity Size Zone (ISZ)	short-zone emphasis, large-zone emphasis, intensity variability, size-zone variability, zone percentage, low-intensity zone emphasis, high-intensity zone emphasis, low-intensity short-zone emphasis, high-intensity short-zone emphasis, low-intensity large-zone emphasis, high-intensity large-zone emphasis
Normalized Co-occurrence (NC)	second angular moment, contrast, entropy, homogeneity, dissimilarity, inverse difference moment, correlation
Voxel Statistics (VS)	minimum SUV, maximum SUV, mean SUV,SUV variance, SUV SD, SUV skewness, SUV kurtosis, SUV skewness (bias corrected), SUV kurtosis (bias corrected), TLG, tumor volume^1^, entropy, SUV _peak_ ^1^
Texture Spectrum (TS)	max spectrum, black-white symmetry
Texture Feature Coding (TFC)	coarseness, homogeneity^1^, mean convergence, variance
Texture Feature Coding Co-occurrence (TFCC)	second angular moment, contrast, entropy, homogeneity, intensity, inverse difference moment, code entropy, code similarity
Neighborhood Gray-Level Dependence (NGLD)	small-number emphasis, large-number emphasis, number non-uniformity, second moment, entropy

^1^These features were not considered in the analyses due to regularity over subsets.

### Statistical analysis

**Fig 2:**
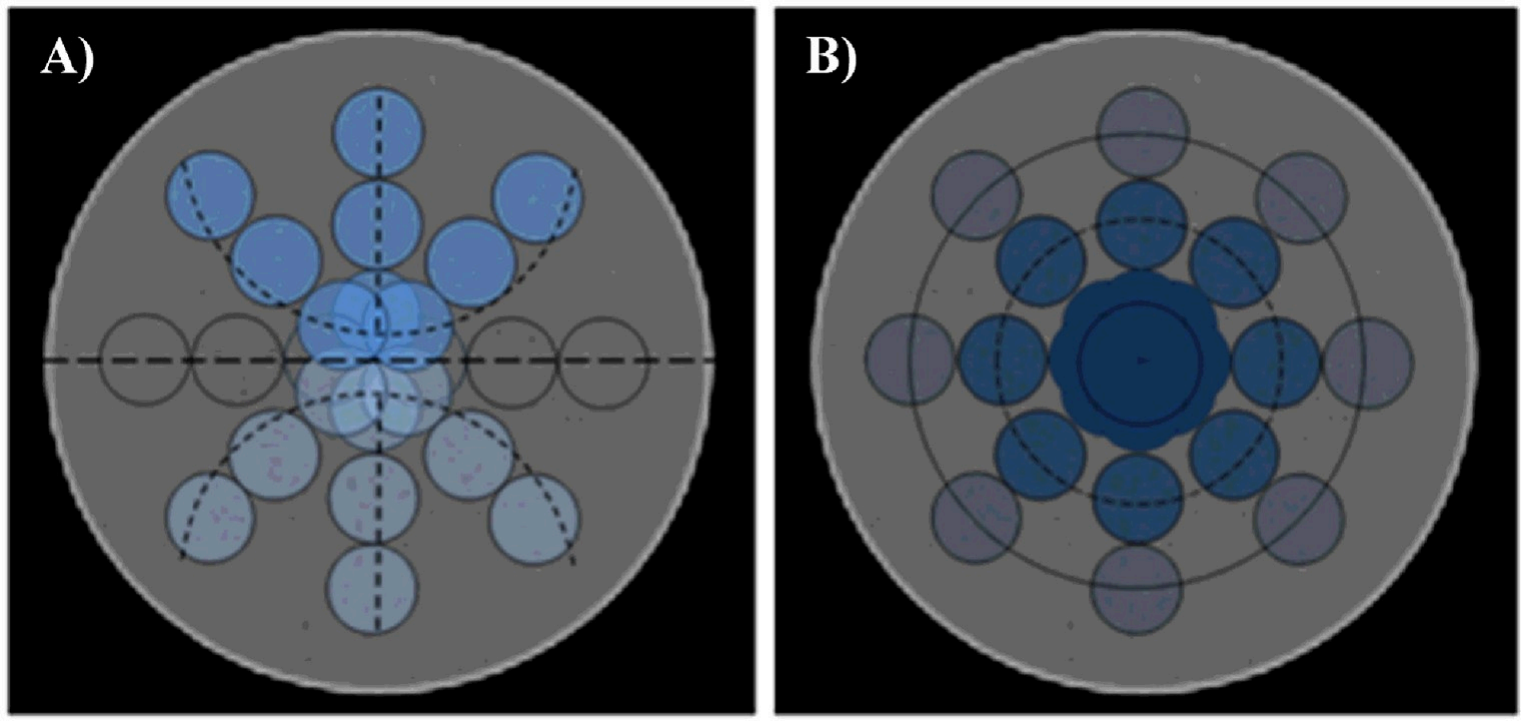
Spatial configurations used for statistical analysis. A) Gravity configuration: the horizontal black dashed line defines the midplane of the phantom, dividing it into two halves. B) Radial configuration: the location of the VOIs is the same as in A), but they are grouped in inner (dotted line), middle (dashed line) and fringe (continuous line) rings. The VOIs crossed by the aforementioned lines were considered for statistical analysis.

The dependence of TA results as a function of the reconstruction parameters was investigated with respect to VOI locations. A total of 13248 (*i*.*e*. 69 texture features x 24 VOIs x 8 SPECT datasets) texture feature values were computed. For each texture feature and fixed VOI location, the feature values were calculated at different SPECT image reconstruction parameters (*i*.*e*. S and I). Moreover, considering that each specific feature could be related to the location of the VOI inside the phantom, they were grouped according to two different spatial configurations, *i*.*e*. 1) gravity and 2) radial configuration, as shown in Fig 2A and 2B, respectively.

The first configuration (Fig 2A) splits the VOIs into two groups with respect to the phantom’s midplane (the ones above and the ones below the horizontal black dashed line in Fig 2A), and each group includes 9 VOIs. The VOIs lying on the phantom’s midplane were omitted as they did not belong to any group. This configuration allowed us to statistically infer whether the texture values were invariant with respect to the symmetry in reference to the midplane.

In the second configuration (Fig 2B), VOIs were grouped to define three different rings (inner, middle and fringe rings), each one including 8 VOIs. This configuration enabled us to statistically test whether the feature values were invariant with respect to VOI location along a radius within the scan field of view.

In the analysis performed we fixed S and considered the texture features as a function of I. The texture feature trend of each VOI represents the datum. Thus, the null hypothesis (H_0_) to be tested was the equality of the average trends grouped according to each VOI spatial configuration (*i*.*e*. gravity and radial). It was evaluated using the non-parametric functional analysis of variance (fANOVA, S1 Appendix). The adopted fANOVA methodology consists in testing H_0_ between projected values of texture feature trends for a given spatial configuration (S1 Appendix) at the significance level (α) of 0.05. In the case of the radial configuration, if H_0_ is rejected, a *post-hoc* analysis (S2 Appendix) is implemented to further investigate the equality of the average trends between VOI groups in two-by-two comparison. With regards to the gravity configuration, the comparison is already between two groups, hence the *post-hoc* analysis may be redundant. Lastly, the cross-correlation coefficients were computed to investigate the relationships between the features that rejected H_0_.

**Fig 3:**
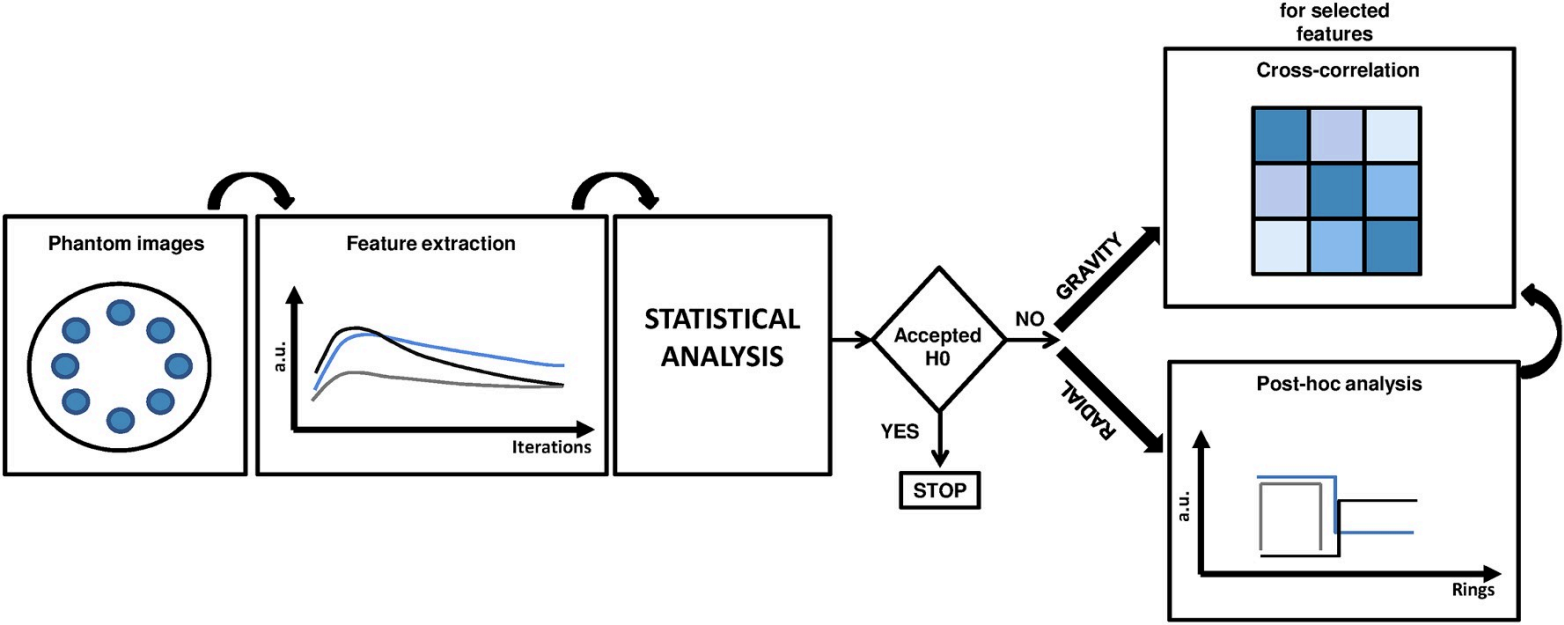
Workflow adopted for the statistical analysis.

Statistical analyses were performed using a script written in the statistical language *R* v.3.4.1 [20]. For the sake of clarity, the workflow adopted for statistical analysis is shown in Fig 3.

## Results

### Gravity configuration

Table 2 shows the corrected *p*-values of the fANOVA test for 5S and 10S performed for the gravity spatial configuration (Fig 2A). For 5S all features reported rejected H_0_ (*i*.*e*. *p*-value < 0.05). However, for 10S no feature rejected H_0_ (a complete report of the corrected *p*-values of the fANOVA test is shown in S1 Table). *Post-hoc* analysis for the gravity configuration was not necessary as the comparison was only among two spatial groups.

**Table 2:** fANOVA results of the gravity configuration. Corrected *p*-values obtained from the statistical analysis on texture features performed for 5S that rejected H_0_. For completeness, the corrected *p*-values for 10S are also reported.

Texture feature	*p*-value	
	5S	10S
NID–strength	0.015	0.428
ISZ–short-zone emphasis	0.028	0.263
VS–minimum SUV	0.002	0.163
TS–black-white symmetry	0.028	0.415
TFCC–intensity	0.007	0.490

### Radial configuration

Tables 3 and 4 show the corrected *p*-values of the fANOVA analysis performed for the radial configuration for 5S and 10S, respectively. Only the texture features that rejected H_0_ are reported in either table (a complete report of the corrected *p*-values of the fANOVA test is shown in S2 Table).

**Table 3:** fANOVA results of the radial configuration for 5S. Corrected *p*-values obtained from the statistical analysis on texture features that rejected H_0_.

Texture feature	*p*-value
	5S
NID–coarseness	0.000
NID–busyness	0.017
NID–strength	0.044
ISZ–intensity variability	0.005
C–correlation	0.002
VS–SUV SD	0.027
VS–SUV kurtosis	0.000
VS–entropy	0.028
TFC–coarseness	0.022
TFCC–second angular moment	0.010
TFCC–entropy	0.008
TFCC–homogeneity	0.008
TFCC–inverse difference moment	0.019
TFCC–code entropy	0.006
TFCC–code similarity	0.003
NGLD–second moment	0.023
NGLD–entropy	0.037

**Table 4:** fANOVA results for the radial configuration for 10S. Corrected *p*-values obtained from the statistical analysis on texture features that rejected H_0_.

Texture feature	*p*-value
	10S
C–homogeneity	0.007
C–inverse difference moment	0.002
VA–run-length variability	0.006
VA–high-intensity short-run emphasis	0.033
NID–coarseness	0.001
NID–busyness	0.014
NID–strength	0.010
ISZ–intensity variability	0.003
NC–homogeneity	0.004
NC–correlation	0.000
VS–SUV kurtosis	0.026
TS–max spectrum	0.027
TFC–coarseness	0.001
TFCC–second angular moment	0.004
TFCC–homogeneity	0.002
TFCC–intensity	0.017
TFCC–inverse difference moment	0.005
TFCC–code entropy	0.001
TFCC–code similarity	0.000
NGLD–number non-uniformity	0.038
NGLD–entropy	0.033

Tables 5 and 6 show the results of *post-hoc* analysis performed to assess the possible two-by-two differences between the average texture feature trends according to the radial grouping (a complete report of the *post-hoc* analysis is shown in S3 and S4 Tables).

**Table 5:** *Post-hoc* analysis results of the radial configuration for 5S.

Texture feature	corrected *p*-value	corrected α	H_0_ rejection
NC–correlation			
inner vs fringe	0.001	0.017	TRUE
middle vs fringe	0.003	0.033	TRUE
inner vs middle	0.074	0.050	FALSE
TFC–coarseness			
inner vs fringe	0.000	0.017	TRUE
middle vs fringe	0.000	0.033	TRUE
inner vs middle	0.166	0.050	FALSE
TFCC–code similarity			
inner vs fringe	0.000	0.017	TRUE
middle vs fringe	0.000	0.033	TRUE
inner vs middle	0.091	0.050	FALSE

TRUE, different average texture feature value between the considered rings; FALSE,equal average texture feature value between the considered rings.

**Table 6:** *Post-hoc* analysis results of the radial configuration for 10S.

Texture feature	corrected *p*-value	corrected α	H_0_ rejection
NID–busyness			
inner vs fringe	0.001	0.017	TRUE
middle vs fringe	0.024	0.033	TRUE
inner vs middle	0.333	0.050	FALSE
TFC–coarseness			
inner vs fringe	0.000	0.017	TRUE
middle vs fringe	0.000	0.033	TRUE
inner vs middle	0.103	0.050	FALSE
TFCC–homogeneity			
inner vs fringe	0.000	0.017	TRUE
middle vs fringe	0.005	0.033	TRUE
inner vs middle	0.196	0.050	FALSE
TFCC–ode ss code similarity			
inner vs fringe	0.000	0.017	TRUE
middle vs fringe	0.010	0.033	TRUE
inner vs middle	0.151	0.050	FALSE

TRUE, different average texture feature value between the considered rings; FALSE,equal average texture feature value between the considered rings.

Texture features NC-correlation, TFC-coarseness and TFCC-code similarity showed a significant difference in average trends inside the rings with respect to 5S. The *post-hoc* analysis identified a radial effect between the fringe ring and both the inner and middle rings, whereas no significant difference was noted between the last two. Indeed, a significant dissimilarity was appreciable at the edge of the phantom (corrected *p*-values refer to inner vs fringe and middle vs fringe). The same radial effect was found for the features NID-busyness, TFC-coarseness, TFCC-homogeneity and TFCC-code similarity for 10S (Table 6).

**Fig 4:**
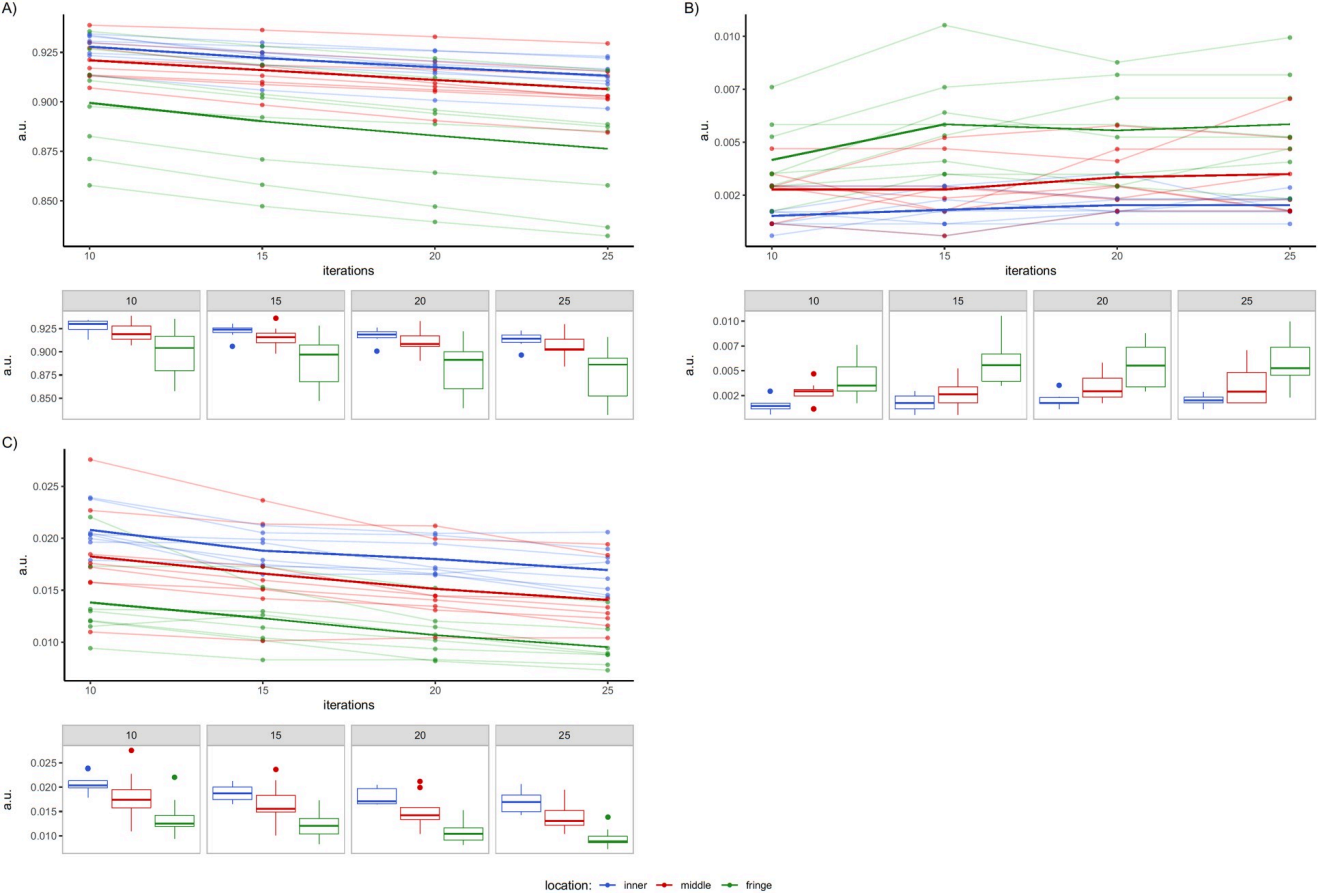
Texture features for VOIs in radial configuration for 5S. A) NC-correlation, B) TFC-coarseness and C) TFCC-code similarity. Dots represent individual texture values inside the VOIs in the considered rings, while thick lines are their average values. Colors indicate spatial groupings of the VOIs (inner, middle and fringe). Box plots are reported under each plot.

**Fig 5:**
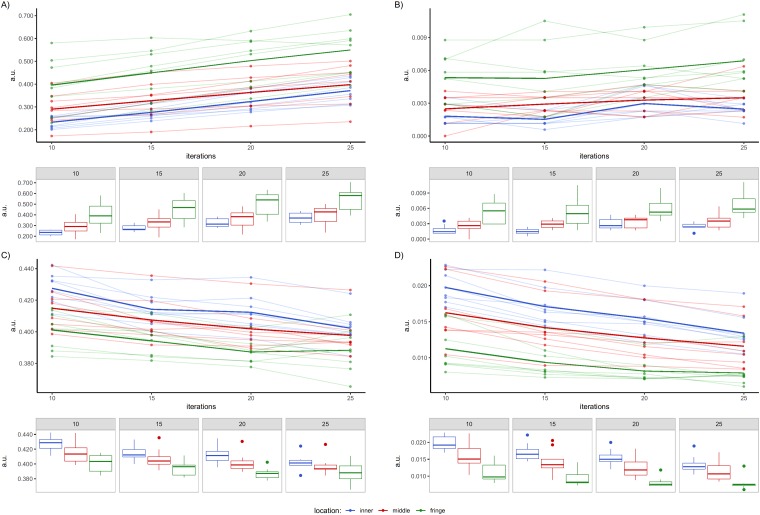
Texture features for VOIs in radial configuration for 10S. A) NID-busyness, B) TFC-coarseness, C) TFCC-homogeneity and D) TFCC-code similarity. Dots represent individual texture values inside the VOIs in the considered rings, while thick lines are their averaged values. Colors indicate spatial groupings of the VOIs (inner, middle and fringe). Box plots are reported under each plot.

A descriptive analysis was also performed of the features that showed in the *post-hoc* analysis a radial effect between both the inner and middle rings with respect to the fringe ring. Specifically, the individual and average trends of such features and the subset-wise cross-correlation between them were graphically investigated. In particular, Fig 4 shows values referring to 5S and the features reported in Table 5, while Fig 5 refers to 10S and the data reported in Table 6. In all figures data are grouped according to VOIs spatial location: points are individual values, whereas thick lines represent the average trend of each ring. Moreover, for sake of clarity, box plots are reported for each ring, I and texture feature shown.

For 5S it is worthy of note that features NC—correlation (Fig 4A) and TFCC–code similarity (Fig 4C) showed similar behaviors for all the rings considered, with texture data for the inner and middle rings higher than that of the fringe ring. This aspect confirms the nature of the features NC-correlation and TFCC-code similarity. Conversely, this relationship was inverted for TFC-coarseness (Fig 4B). In particular, NC—correlation quantifies the linear dependency of grey levels in neighborhood pixels with a specific distance between pixels (in our case, 1 pixel) and angular orientation (averaged in our case), while TFCC-code similarity was calculated using the TFC method. This coding approach transforms the original image into a texture feature image in which each pixel value is a Texture Feature Number (TFN). In particular, for a texture image pixel, the TFN is generated on the basis of the grey-level changes in the surrounding pixels, which represent the texture unit. Successively, the TFC—co-occurrence matrix is calculated starting from the texture image, and represents the local variation of the TFNs. The same approach is used to calculate the TFC—coarseness, this last feature representing a drastic change in the TFNs of the texture unit.

For 10S, NID-busyness (Fig 5A), TFC-coarseness (Fig 5B), TFCC-homogeneity (Fig 5C) and TFCC-code similarity (Fig 5D) rejected H_0_. TFCC-homogeneity represents no significant changes in the TFNs of the texture unit, and it had the same trend as TFCC-code similarity, with higher values for the inner and middle locations. NID-busyness represents the spatial frequency of changes in grey-level intensity between one pixel and its neighbors, and it had a similar trend to that of TFC-coarseness, with higher average trajectory for the fringe ring. However, the trend was inverted with respect to Fig 5C and 5D.

**Fig 6:**
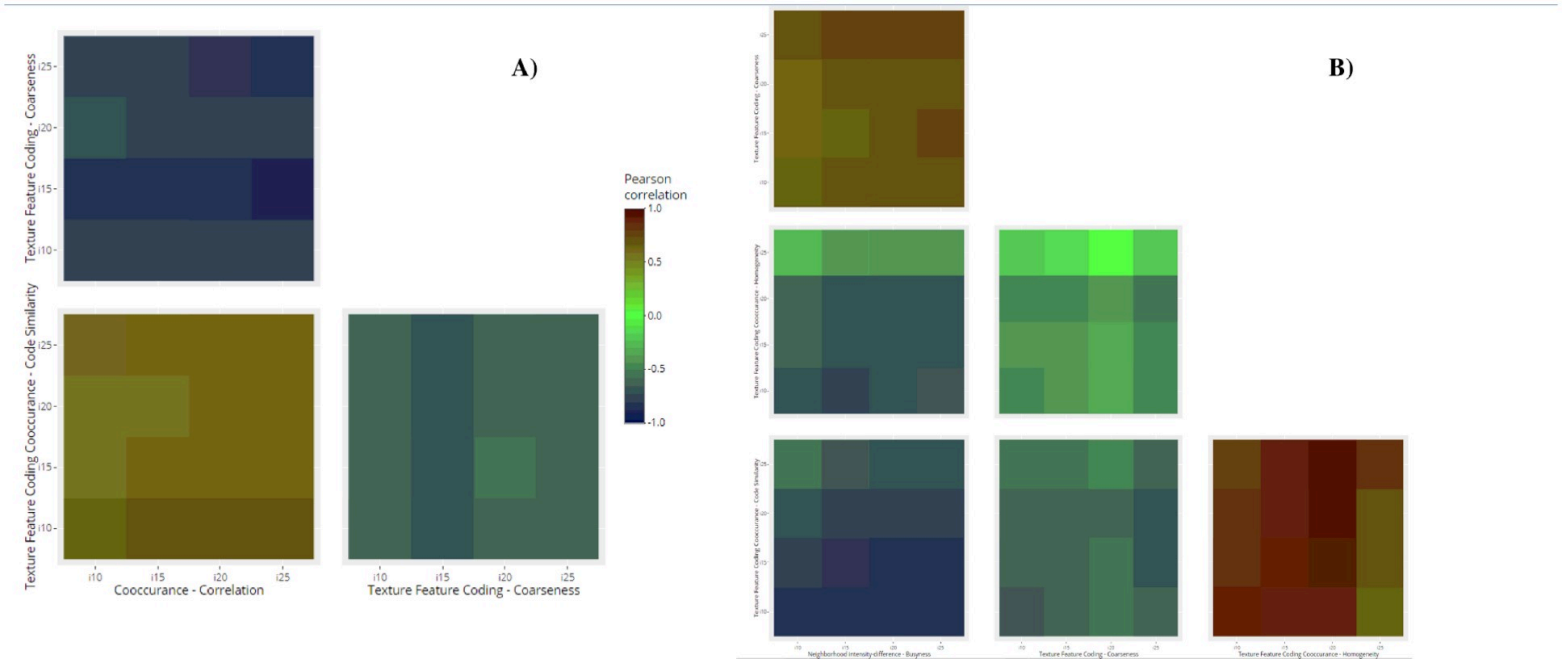
Heat-maps of cross-correlation coefficients between texture features rejecting H_0_ in the radial configuration. In each heat-map the horizontal and vertical axes refer to the value of the texture features obtained at a specific I for A) 5S and B) 10S. The legend reported in both maps refers to the Pearson’s correlation coefficient, and the color associated with a legend value is reported on the square regions of the maps, representing the level of correlation between the texture features.

Finally, Fig 6 shows the heat-maps of the cross-correlation between those texture features showing significant differences among fringe and both middle and inner rings in the *post-hoc* analysis. With regard to 5S (Fig 6A), TFC-coarseness negatively cross-correlated with the other two features (*i*.*e*. NC-correlation and TFCC-similarity) once more highlighting the opposite aspects they measure. Conversely, NC-correlation and TFCC-code similarity showed a positive cross-correlation. Similarly, NID-busyness vs TFC-coarseness and TFCC-homogeneity vs TFCC-similarity for 10S (Fig 6B) had high positive cross-correlation values, confirming their trends in Fig 5A and 5B. The cross-correlation is negative for the remaining features.

## Discussions

SPECT images consist of a sequence of slice images containing information about the distribution of the radiotracer in form of counts stored in each pixel. Although some commercial SPECT systems show comparable performances [21], the reconstruction artifacts remain a handicap towards accurate quantification in SPECT acquisitions. International protocols provide methods for data quantification and comparison of SPECT images [19, 20], proposing that the spatial information of the radiotracer distribution at the pixel level be mediated over the neighboring pixels. Conversely, TA provides methods to re-introduce this spatial information at pixel level by means of heterogeneity quantification. Recently, TA approach has been proven to be suitable for quantitative assessment of the quality assurance procedures based on visual inspection in SPECT systems [13]. With regard to tomographic uniformity, an international protocol [18] proposed a quantitative evaluation limited to a 15x15-pixel square ROI centered on several reconstructed slices, whereas judgment about the overall tomographic uniformity of the phantom actually pertained to the visual assessment. With this in mind, we used TA on reconstructed ^177^Lu SPECT images of homogeneous phantom to address the impact of reconstruction algorithm. The trend of different texture features as a function of 3D-OSEM parameters (*i*.*e*. S and I) was investigated for different VOI location to identify non–uniform regions and to underline potential discrepancies between them. We did this for two reasons: 1) to overcome the limitations of the visual assessment of SPECT uniformity evaluation and 2) to study non-uniformities, which are of particular interest because any non-uniformity in a SPECT system is amplified by the tomographic reconstruction process. The latter reason is even more important as recent publications have proposed using a uniform phantom for SPECT calibration [17, 22–24], within the context of personalized dosimetry for molecular radiotherapy (MRT) [25, 26].

In the present study, two spatial configurations (*i*.*e*. gravity and radial) were used to investigate: 1) the isotropy of the SPECT signal produced by the ^177^Lu activity distribution and 2) the influence of reconstruction parameters and VOI location (Fig 3) on texture features. We also used VOIs rather than ROIs, as suggested by the international protocols on SPECT image quality evaluation [18].

With regard to gravity configuration, a visual inspection of the reconstructed SPECT images shown in Fig 1E (5S) and Fig 1F (10S) reveals the presence of blobby noise due to the reconstruction parameters used. Fixing S, the noise increased as I increased, and this also occurred when I was fixed but S increased [23]. The increase in noise in the considered images is also confirmed by the line profile rippling along the dotted lines in the reconstructed SPECT images (supplementary materials S1 Fig). In particular, the profiles obtained showed two pronounced horns on each side with respect to their central region due to the use of CDR in the SPECT reconstruction process, as reported in [14].

The TA results in Table 2 relating to gravity configuration show some exceptions for 5S, suggesting non-uniformity inside the SPECT images. These findings disappear at 10S (Table 2) for which all texture features investigated were invariant with respect to the symmetry relating to the midplane of the phantom image (*i*.*e*. equality of average texture trend between the two halves). It can be assumed that the effect observed for 5S was a consequence of the 3D-OSEM reconstruction algorithm used: as S increased, so did the number of projections used in the reconstruction process. Given that counts provide information on ^177^Lu radioactivity at pixel level, the results obtained in the present study indicate that S<10 do not provide reliable information on the uniformity requirements for SPECT images due to the effect of the reconstruction algorithm. These findings are in agreement with the results reported in [18], where S = 10 was considered the best compromise between reducing the noise in the reconstructed images and diminishing difference between reconstructed and real activity. For this reason no cross-correlation analysis was performed for S = 5.

With regard to the radial configuration, Tables 3 and 4 summarize the texture features that rejected H_0_ (*i*.*e*. at least one spatial grouping average texture trend differed from the others), moving from the center of the reconstructed SPECT image towards its edge. Rejection of H_0_ is directly related to the reconstruction algorithm: the contribution of a given pixel is related to its position inside the imaged phantom and to SPECT acquisition angle [27]. Among these features, there were thirteen common features rejecting H_0_ (features reported in bold in Tables 3 and 4) for both 5S and 10S, revealing differences between the edge of the phantom and the inner and middle rings. *Post-hoc* analysis identified common features (Table 5 for 5S, and Table 6 for 10S) presenting differences between the three rings considered. SPECT uniformity was assured in the inner and middle rings, as detected by second order statistics. The first order statistics (*i*.*e*. Voxel Statistics) did not reject H_0_ (as reported in Tables 3 and 4), but the *post-hoc* analysis revealed that it was not suitable to detect non-uniformities inside the phantom. Moreover, the approach based on a comparison of the rings not only confirmed the uniformity in the central region but also provided information on its extension and, consequently, on the phantom portion suitable for SPECT calibration.

The texture features selected by the *post-hoc* analysis are capable of detecting non-uniformities, and they could provide a new key lecture for the image quality assessment. Moreover, this investigation attempts to overcome the limitation declared in a published study [13], with the aim to address quantitatively the possible non-uniformity sources due to reconstruction process. However, our results must now be validated by extending the present investigation to different gamma camera systems and related reconstruction workstations, at the same time considering the role of post filtering parameters at the end of the reconstruction process.

## Conclusions

In the present study we investigated the uniformity of the reconstructed SPECT images of a homogeneous phantom using TA. In particular, statistical analysis revealed that some texture features are indicative of differences between VOIs in the reconstructed SPECT images, providing a new “finger-print” in the assessment of quality assurance for SPECT systems. Future developments of this study include further validations of different gamma camera systems and reconstruction workstations with the aim of defining a new metric based on TA methods.

## Supporting information

S1 Appendix (DOCX)

S2 Appendix (DOCX)

S1 Table fANOVA results for the gravity configuration. Corrected p-values for all texture features considered in the study obtained from the statistical analysis performed for the gravity configuration for 5S and 10S. The bold values reported only for the case of 5 subsets indicate that the texture feature violates the null hypothesis (i.e. equality of the average texture feature trends related to the VOIs in the above and below the phantom’s midplane).
(DOCX)

S2 Table fANOVA results for the radial configuration. Corrected p-values for all texture features considered in the study obtained from the statistical analysis performed for the radial configuration for 5S and 10S. The bold values reported indicate that the texture feature violates the null hypothesis of equality of the average texture feature trends in the region considered (i.e. inner, middle and fringe).
(DOCX)

S3 Table Post-hoc analysis for the radial configuration for 5S. All texture features here reported are the same reported in Table 3, and the bold values indicate those texture features that violates the null hypothesis of the post-hoc analysis.
(DOCX)

S4 Table Post-hoc analysis for the radial configuration for 10 subsets. All texture features here reported are the same reported in Table 4, and the bold values indicate those texture features that violates the null hypothesis of the post-hoc analysis.
(DOCX)

S1 Fig Noise profile on reconstructed SPECT images. Reconstructed images of the homogeneous phantom and line profile of pixel counts. A) Central slice of the homogeneous phantom reconstructed with different number of subsets and iterations (reported under each image). B) Counts profile along the dashed yellow line in A) considered for the two datasets. C) Counts profile for 5S1I and 10S1I along the yellow line in A).
(DOCX)
