# Supplementary material for: Texture analysis in 177Lu SPECT phantom images: Statistical assessment of uniformity requirements using texture features
Source: PLoS One. 2019 Jul 31;14(7):e0218814. doi: 10.1371/journal.pone.0218814 (PMC6668785; doi:10.1371/journal.pone.0218814)
Supplement: S1 Appendix — (DOCX) [file pone.0218814.s001.docx]

**S1 Appendix.**

**Functional analysis of variance (fANOVA)**

Functional data analysis is a branch of statistics where the datasets are functions instead of data points. The comparison of average trends from different groups requires techniques able to exploit the complex dependence of each datum (*i.e*. function) as a function of the same set of values. In our case, the function is each texture feature considered in a VOI located inside the phantom, and the values are the OSEM parameters (*i.e*. number of subsets and iterations) considered in the study.

For each texture feature, let:

$y_{ij}^{s}=\left( y_{1j}^{s},\ldots,y_{kj}^{s} \right)$ (A1)

be the $k$-dimensional vector, whose components represent the *j-th* VOI’s texture feature (*j=1,…,n*) as a function of the number of iteration *i* (*i=1,…,k*) for a fixed number of subset *s* (*s=*5;10). Furthermore, we state a *k x n* matrix:

$Z^{s}=(z_{1j}^{s},\ldots,z_{kj}^{s})$ (A2)

where each element is a rescaled VOI’s texture feature, defined as:

$z_{ij}^{s}=\frac{y_{ij}^{s}-min(y_{ij}^{s})}{\max\left( y_{ij}^{s} \right)-min(y_{ij}^{s})}$ (A3)

Finally, let $\tilde{Z^{\boldsymbol{s}}}$ be the Mahalanobis transformed matrix (MTM) of the rescaled VOI’s texture feature matrix $Z^{s}$, having uncorrelated columns [1]. Using a novel approach to fANOVA [2], each column of the $\tilde{Z^{s}}$ matrix is transformed into a synthetic value, named projected value ($p_{j}^{s}$) defined as follows:

$p_{j}^{s}=\sum_{i=1}^{k} Y_{ij}\cdot\tilde{z_{ij}^{s}}$ (A4)

.where:

$\tilde{z_{ij}^{s}}$_ is *i-th* element of the *j-th* column of the $\tilde{Z^{\boldsymbol{s}}}$ matrix, and $Y_{ij}$ random number generated from independent and identically distributed (i.i.d.) normal distributions.

As the projected values $p_{j}^{s}$ do not verify the usual ANOVA assumption (*i.e*. normality of the data distribution and homoscedasticity), a novel non-parametric approach [3] was used. Based on this approach, the $p_{j}^{s}$ can be used to statistically infer the null hypothesis of equality of the average texture feature trends according to each VOI spatial configuration (i.e. gravity and radial)., setting the significance level (α) at 0.05.

The randomness induced by generating the $Y_{ij}$ numbers can be managed by independently repeating several times the projection procedure and the statistical test. However, some sort of control on the false discovery rate (FDR) and a way of quantifying the number of repetitions have to be included. For these tasks, the procedure adopted and reported in [1] consists on repeating the analysis as many times as the dimension *k* of the values (*i.e*. the number of iterations). Moreover, in order to reduce the FDR induced by multiple testing the *k* different random projections of the same data (*i.e*. $\tilde{z_{ij}^{s}}$), the correction of the *p*-value [4] is used, allowing at most to reject the null hypothesis at the nominal significance level (*i.e.* α = 0.05).

**References**

1. Mardia KV, Kent JT and Bibby JM. Multivariate analysis. Academic Press. 1979.

2. Cuesta Albertos JA and Febrero-Bande M. A simple multiway ANOVA for functional data. Test. 2010. doi: 10.1007/s11749-010-0185-3.

3. Brunner E, Konietschke F, Pauly M and Puri ML. Rank-based procedures in factorial design: hypothesis about non parametric treatments effects. Journal of the Royal Statistical Society.2017. doi:10.12/aos/1013699998

4. Benjiamini Y and Yekutieli D. The control of the false discovery rate in multiple testing under dependency. Annals of Statistics. 2001. doi:10.1214/aos/1013699998
