# Supplementary material for: Texture analysis in 177Lu SPECT phantom images: Statistical assessment of uniformity requirements using texture features
Source: PLoS One. 2019 Jul 31;14(7):e0218814. doi: 10.1371/journal.pone.0218814 (PMC6668785; doi:10.1371/journal.pone.0218814)
Supplement: S2 Appendix — (DOCX) [file pone.0218814.s002.docx]

**S2 Appendix.**

***Post-hoc* analysis**

In case of rejection of the fANOVA null hypothesis (H_0_) of the equality of average trends for three or more groups, a further investigation on the causes of such violation could be performed in a two-by-two comparison. Several tests have been proposed in the literature for a standard analysis (i.e. univariate normal distributed and homoscedastic data) in order to control the familywise error rate (*e.g.* Tukey test [1]). However, given the complexity of functional data, no standard analysis is available, and a novel approach can be taken into account that tests the H_0_ for all the possible dyads. Thus, a control of the false discovery rate (FDR) has to be included to prevent an inflation of the type I error rate.

With this in mind, in the case of rejection of the H_0_ of equality of the average texture feature trends by fANOVA in the radial configuration, a *post-hoc* analysis was performed with the aim to investigate which spatial grouping of the VOIs (*i.e*. inner, middle, fringe) could possibly cause this violation. The new hypothesis is based on a two-by-two comparison of the average VOI group trends (*i.e*. inner versus middle; inner versus fringe; middle versus fringe).. The same fANOVA procedure was retained. However, due the repetition of the testing procedure on the same data, the Benjamini and Yekutieli correction of the *p*-values [2] has been adopted to reduce the FDR.

It is worth stressing that for the gravity configuration the *post-hoc* analysis was not necessary as only two spatial groupings are compared.

**References**

1. Tukey, J. Comparing Individual Means in the Analysis of Variance. Biometrics. 1949. doi: 10.2307/3001913

2. Benjiamini Y and Yekutieli D. The control of the false discovery rate in multiple testing under dependency. Annals of Statistics. 2001. doi:10.1214/aos/1013699998
