# Supplementary material for: Texture analysis in 177Lu SPECT phantom images: Statistical assessment of uniformity requirements using texture features
Source: PLoS One. 2019 Jul 31;14(7):e0218814. doi: 10.1371/journal.pone.0218814 (PMC6668785; doi:10.1371/journal.pone.0218814)
Supplement: S2 Table — Corrected p-values for all texture features considered in the study obtained from the statistical analysis performed for the radial configuration for 5S and 10S. The bold values reported indicate that the texture feature violates the null hypothesis of equality of the average texture feature trends in the region considered (i.e. inner, middle and fringe). (DOCX) [file pone.0218814.s004.docx]

| **Texture feature** | ***p*-values** | |
| --- | --- | --- |
|  | **5S** | **10S** |
| Cooccurance - second angular moment | 0,416 | 0,102 |
| Cooccurance - contrast | 0,537 | 0,063 |
| Cooccurance - entropy | 0,814 | 0,219 |
| Cooccurance - homogeneity | 0,729 | **0,007** |
| Cooccurance - dissimilarity | 0,478 | 0,095 |
| Cooccurance - inverse difference moment | 0,653 | **0,002** |
| Voxel Alignment - short run emphasis | 0,266 | 0,076 |
| Voxel Alignment - long run emphasis | 0,083 | 0,690 |
| Voxel Alignment - intensity variability | 0,839 | 0,428 |
| Voxel Alignment - run-length variability | 0,196 | **0,006** |
| Voxel Alignment - run percentage | 0,063 | 0,111 |
| Voxel Alignment - low-intensity run emphasis | 0,207 | 0,716 |
| Voxel Alignment - high-intensity run emphasis | 0,709 | 0,190 |
| Voxel Alignment - low-intensity short-run emphasis | 0,905 | 0,315 |
| Voxel Alignment - high-intensity short-run emphasis | 0,973 | **0,033** |
| Voxel Alignment - low-intensity long-run emphasis | 0,610 | 0,884 |
| Voxel Alignment - high-intensity long-run emphasis | 0,389 | 0,692 |
| Neighborhood Intensity Difference - coarseness | **0,000** | **0,001** |
| Neighborhood Intensity Difference - contrast | 0,453 | 0,123 |
| Neighborhood Intensity Difference - busyness | **0,017** | **0,014** |
| Neighborhood Intensity Difference - complexity | 0,224 | 0,460 |
| Neighborhood Intensity Difference - strength | **0,044** | **0,010** |
| Intensity Size Zone - short-zone emphasis | 0,915 | 0,556 |
| Intensity Size Zone - large-zone emphasis | 0,214 | 0,148 |
| Intensity Size Zone - intensity variability | **0,005** | **0,003** |
| Intensity Size Zone - size-zone variability | 0,267 | 0,602 |
| Intensity Size Zone - zone percentage | 0,425 | 0,105 |
| Intensity Size Zone - low-intensity zone emphasis | 0,105 | 0,162 |
| Intensity Size Zone - high-intensity zone emphasis | 0,936 | 0,106 |
| Intensity Size Zone - low-intensity short-zone emphasis | 0,905 | 0,697 |
| Intensity Size Zone - high-intensity short-zone emphasis | 0,478 | 0,504 |
| Intensity Size Zone - low-intensity large-zone emphasis | 0,526 | 0,181 |
| Intensity Size Zone - high-intensity large-zone emphasis | 0,070 | 0,480 |
| Normalized Cooccurance - second angular moment | 0,870 | 0,120 |
| Normalized Cooccurance - contrast | 0,325 | 0,293 |
| Normalized Cooccurance - entropy | 0,787 | 0,440 |
| Normalized Cooccurance - homogeneity | 0,551 | **0,004** |
| Normalized Cooccurance - dissimilarity | 0,450 | 0,132 |
| Normalized Cooccurance - inverse difference moment | 0,670 | 0,090 |
| Normalized Cooccurance - correlation | **0,002** | **0,000** |
| Voxel Statisticss - minimum SUV | 0,764 | 0,532 |
| Voxel Statistics - maximum SUV | 0,228 | 0,141 |
| Voxel Statistics - mean SUV | 0,170 | 0,131 |
| Voxel Statistics - SUV variance | 0,183 | 0,069 |
| Voxel Statistics - SUV SD | **0,027** | 0,079 |
| Voxel Statistics - SUV skewness | 0,782 | 0,154 |
| Voxel Statistics - SUV kurtosis | **0,000** | **0,026** |
| Voxel Statistics - SUV bias-corrected skewness | 0,993 | 0,485 |
| Voxel Statistics - SUV bias-corrected kurtosis | 0,301 | 0,132 |
| Voxel Statistics - TLG | 0,658 | 0,161 |
| Voxel Statistics - entropy | **0,028** | 0,773 |
| Texture Spectrum - max spectrum | 0,264 | **0,027** |
| Texture Spectrum - black-white symmetry | 0,953 | 0,449 |
| Texture Feature Coding - coarseness | **0,022** | **0,001** |
| Texture Feature Coding - mean convergence | 0,780 | 0,375 |
| Texture Feature Coding - variance | 0,971 | 0,872 |
| Texture Feature Coding Cooccurance - second angular moment | **0,010** | **0,004** |
| Texture Feature Coding Cooccurance - contrast | 0,729 | 0,789 |
| Texture Feature Coding Cooccurance - entropy | **0,008** | 0,096 |
| Texture Feature Coding Cooccurance - homogeneity | **0,008** | **0,002** |
| Texture Feature Coding Cooccurance - intensity | 0,320 | **0,017** |
| Texture Feature Coding Cooccurance - inverse difference moment | **0,019** | **0,005** |
| Texture Feature Coding Cooccurance - code entropy | **0,006** | **0,001** |
| Texture Feature Coding Cooccurance - code similarity | **0,003** | **0,000** |
| Neighboring Gray Level Dependence - small number emphasis | 0,188 | 0,055 |
| Neighboring Gray Level Dependence - large number emphasis | 0,367 | 0,195 |
| Neighboring Gray Level Dependence - number non-uniformity | 0,306 | **0,038** |
| Neighboring Gray Level Dependence - second moment | **0,023** | 0,058 |
| Neighboring Gray Level Dependence - entropy | **0,037** | **0,033** |

**S2 Table. fANOVA results for the radial configuration.** Corrected *p*-values for all texture features considered in the study obtained from the statistical analysis performed for the radial configuration for 5S and 10S. The bold values reported indicate that the texture feature violates the null hypothesis of equality of the average texture feature trends in the region considered (*i.e*. inner, middle and fringe).
