# Supplementary material for: Texture analysis in 177Lu SPECT phantom images: Statistical assessment of uniformity requirements using texture features
Source: PLoS One. 2019 Jul 31;14(7):e0218814. doi: 10.1371/journal.pone.0218814 (PMC6668785; doi:10.1371/journal.pone.0218814)
Supplement: S3 Table — All texture features here reported are the same reported in Table 3, and the bold values indicate those texture features that violates the null hypothesis of the post-hoc analysis. (DOCX) [file pone.0218814.s005.docx]

| **Parent matrix - Texture feature** | **Corrected**  **p-value** | **Corrected α** | **Rejection of null hypothesis** |
| --- | --- | --- | --- |
|  |  |  |  |
|  |  |  |  |
|  |  |  |  |
| Neighborhood Intensity Difference - coarseness |  |  |  |
| inner vs fringe | 0,000 | 0,017 | TRUE |
| middle vs fringe | 0,055 | 0,050 | FALSE |
| inner vs middle | 0,010 | 0,033 | TRUE |
| Neighborhood Intensity Difference - busyness |  |  |  |
| inner vs fringe | 0,039 | 0,033 | FALSE |
| middle vs fringe | 0,154 | 0,050 | FALSE |
| inner vs middle | 0,003 | 0,017 | TRUE |
| Neighborhood Intensity Difference - strength |  |  |  |
| inner vs fringe | 0,003 | 0,017 | TRUE |
| middle vs fringe | 0,111 | 0,033 | FALSE |
| inner vs middle | 0,278 | 0,050 | FALSE |
| Intensity Size Zone - intensity variability |  |  |  |
| inner vs fringe | 0,036 | 0,017 | FALSE |
| middle vs fringe | 0,130 | 0,050 | FALSE |
| inner vs middle | 0,054 | 0,033 | FALSE |
| **Normalized Cooccurance - correlation** |  |  |  |
| **inner vs fringe** | **0,003** | **0,033** | **TRUE** |
| **middle vs fringe** | **0,001** | **0,017** | **TRUE** |
| **inner vs middle** | **0,074** | **0,050** | **FALSE** |
| Voxel Statistics - SUV SD |  |  |  |
| inner vs fringe | 0,101 | 0,033 | FALSE |
| middle vs fringe | 0,037 | 0,017 | FALSE |
| inner vs middle | 0,169 | 0,050 | FALSE |
| Voxel Statistics - SUV kurtosis |  |  |  |
| inner vs fringe | 0,000 | 0,017 | TRUE |
| middle vs fringe | 0,106 | 0,033 | FALSE |
| inner vs middle | 0,488 | 0,050 | FALSE |
| Voxel Statistics - entropy |  |  |  |
| inner vs fringe | 0,020 | 0,033 | TRUE |
| middle vs fringe | 0,699 | 0,050 | FALSE |
| inner vs middle | 0,000 | 0,017 | TRUE |
| **Texture Feature Coding - coarseness** |  |  |  |
| **inner vs fringe** | **0,000** | **0,017** | **TRUE** |
| **middle vs fringe** | **0,000** | **0,033** | **TRUE** |
| **inner vs middle** | **0,166** | **0,050** | **FALSE** |
| Texture Feature Coding Cooccurance - second angular moment |  |  |  |
| inner vs fringe | 0,000 | 0,017 | TRUE |
| middle vs fringe | 0,562 | 0,050 | FALSE |
| inner vs middle | 0,089 | 0,033 | FALSE |
| Texture Feature Coding Cooccurance - entropy |  |  |  |
| inner vs fringe | 0,023 | 0,017 | FALSE |
| middle vs fringe | 0,699 | 0,050 | FALSE |
| inner vs middle | 0,315 | 0,033 | FALSE |
| Texture Feature Coding Cooccurance - homogeneity |  |  |  |
| inner vs fringe | 0,000 | 0,017 | TRUE |
| middle vs fringe | 0,034 | 0,033 | FALSE |
| inner vs middle | 0,154 | 0,050 | FALSE |
| Texture Feature Coding Cooccurance - inverse difference moment |  |  |  |
| inner vs fringe | 0,000 | 0,017 | TRUE |
| middle vs fringe | 0,146 | 0,033 | FALSE |
| inner vs middle | 0,260 | 0,050 | FALSE |
| Texture Feature Coding Cooccurance - code entropy |  |  |  |
| inner vs fringe | 0,000 | 0,017 | TRUE |
| middle vs fringe | 0,265 | 0,050 | FALSE |
| inner vs middle | 0,148 | 0,033 | FALSE |
| **Texture Feature Coding Cooccurance - code similarity** |  |  |  |
| **inner vs fringe** | **0,000** | **0,017** | **TRUE** |
| **middle vs fringe** | **0,000** | **0,033** | **TRUE** |
| **inner vs middle** | **0,091** | **0,050** | **FALSE** |
| Neighboring Gray Level Dependence - second moment |  |  |  |
| inner vs fringe | 0,091 | 0,033 | FALSE |
| middle vs fringe | 0,037 | 0,017 | FALSE |
| inner vs middle | 0,849 | 0,050 | FALSE |
| Neighboring Gray Level Dependence - entropy |  |  |  |
| inner vs fringe | 0,049 | 0,033 | FALSE |
| middle vs fringe | 0,188 | 0,050 | FALSE |
| inner vs middle | 0,000 | 0,017 | TRUE |

**S3 Table. *Post-hoc* analysis for the radial configuration for 5S.**  All texture features here reported are the same reported in table 3, and the bold values indicate those texture features that violates the null hypothesis of the *post-hoc* analysis.
