# Supplementary material for: Texture analysis in 177Lu SPECT phantom images: Statistical assessment of uniformity requirements using texture features
Source: PLoS One. 2019 Jul 31;14(7):e0218814. doi: 10.1371/journal.pone.0218814 (PMC6668785; doi:10.1371/journal.pone.0218814)
Supplement: S4 Table — All texture features here reported are the same reported in Table 4, and the bold values indicate those texture features that violates the null hypothesis of the post-hoc analysis. (DOCX) [file pone.0218814.s006.docx]

| **Parent matrix - Texture feature** | **Corrected**  **p-value** | **Corrected α** | **H_0_ rejection** |
| --- | --- | --- | --- |
|  |  |  |  |
|  |  |  |  |
| Cooccurance - homogeneity |  |  |  |
| inner vs fringe | 0,000 | 0,017 | TRUE |
| middle vs fringe | 0,502 | 0,050 | FALSE |
| inner vs middle | 0,006 | 0,033 | TRUE |
| Cooccurance - inverse difference moment |  |  |  |
| inner vs fringe | 0,000 | 0,017 | TRUE |
| middle vs fringe | 0,696 | 0,050 | FALSE |
| inner vs middle | 0,000 | 0,033 | TRUE |
| Voxel alignment - run length variability |  |  |  |
| inner vs fringe | 0,001 | 0,017 | TRUE |
| middle vs fringe | 0,096 | 0,033 | FALSE |
| inner vs middle | 0,205 | 0,050 | FALSE |
| Voxel alignment - high intensity short run emphasis |  |  |  |
| inner vs fringe | 0,134 | 0,033 | FALSE |
| middle vs fringe | 0,655 | 0,050 | FALSE |
| inner vs middle | 0,086 | 0,017 | FALSE |
| Neighborhood Intensity Difference - coarseness |  |  |  |
| inner vs fringe | 0,000 | 0,017 | TRUE |
| middle vs fringe | 0,023 | 0,050 | TRUE |
| inner vs middle | 0,000 | 0,033 | TRUE |
| **Neighborhood Intensity Difference - busyness** |  |  |  |
| **inner vs fringe** | **0,001** | **0,017** | **TRUE** |
| **middle vs fringe** | **0,024** | **0,033** | **TRUE** |
| **inner vs middle** | **0,333** | **0,050** | **FALSE** |
| Neighborhood Intensity Difference - strength |  |  |  |
| inner vs fringe | 0,000 | 0,017 | TRUE |
| middle vs fringe | 0,246 | 0,050 | FALSE |
| inner vs middle | 0,031 | 0,033 | TRUE |
| Intensity Size Zone - intensity variability |  |  |  |
| inner vs fringe | 0,095 | 0,033 | FALSE |
| middle vs fringe | 0,074 | 0,017 | FALSE |
| inner vs middle | 0,738 | 0,050 | FALSE |
| Normalized Cooccurance - homogeneity |  |  |  |
| inner vs fringe | 0,020 | 0,017 | FALSE |
| middle vs fringe | 0,591 | 0,050 | FALSE |
| inner vs middle | 0,101 | 0,033 | FALSE |
| Normalized Cooccurance - correlation |  |  |  |
| inner vs fringe | 0,000 | 0,017 | TRUE |
| middle vs fringe | 0,070 | 0,050 | FALSE |
| inner vs middle | 0,055 | 0,033 | FALSE |
| Voxel Statistics - SUV.kurtosis |  |  |  |
| inner vs fringe | 0,069 | 0,033 | FALSE |
| middle vs fringe | 0,772 | 0,050 | FALSE |
| inner vs middle | 0,013 | 0,017 | TRUE |
| Texture Spectrum - max spectrum |  |  |  |
| inner vs fringe | 0,504 | 0,050 | FALSE |
| middle vs fringe | 0,023 | 0,033 | TRUE |
| inner vs middle | 0,001 | 0,017 | TRUE |
| **Texture Feature Coding - coarseness** |  |  |  |
| **inner vs fringe** | **0,000** | **0,017** | **TRUE** |
| **middle vs fringe** | **0,000** | **0,033** | **TRUE** |
| **inner vs middle** | **0,103** | **0,050** | **FALSE** |
| Texture Feature Coding Cooccurance - second angular moment |  |  |  |
| inner vs fringe | 0,003 | 0,017 | TRUE |
| middle vs fringe | 0,277 | 0,033 | FALSE |
| inner vs middle | 0,407 | 0,050 | FALSE |
| **Texture Feature Coding Cooccurance - homogeneity** |  |  |  |
| **inner vs fringe** | **0,000** | **0,017** | **TRUE** |
| **middle vs fringe** | **0,005** | **0,033** | **TRUE** |
| **inner vs middle** | **0,196** | **0,050** | **FALSE** |
| Texture Feature Coding Cooccurance - intensity |  |  |  |
| inner vs fringe | 0,296 | 0,017 | FALSE |
| middle vs fringe | 0,309 | 0,033 | FALSE |
| inner vs middle | 0,557 | 0,050 | FALSE |
| Texture Feature Coding Cooccurance - inverse difference moment |  |  |  |
| inner vs fringe | 0,003 | 0,017 | TRUE |
| inner vs middle | 0,482 | 0,033 | FALSE |
| middle vs fringe | 0,498 | 0,050 | FALSE |
| Texture Feature Coding Cooccurance - code entropy |  |  |  |
| inner vs fringe | 0,005 | 0,017 | TRUE |
| inner vs middle | 0,275 | 0,033 | FALSE |
| middle vs fringe | 0,482 | 0,050 | FALSE |
| **Texture Feature Coding Cooccurance - code similarity** |  |  |  |
| **inner vs fringe** | **0,000** | **0,017** | **TRUE** |
| **middle vs fringe** | **0,010** | **0,033** | **TRUE** |
| **inner vs middle** | **0,151** | **0,050** | **FALSE** |
| Neighboring Gray Level Dependence - number nonuniformity |  |  |  |
| inner vs fringe | 0,020 | 0,017 | FALSE |
| middle vs fringe | 0,799 | 0,050 | FALSE |
| inner vs middle | 0,052 | 0,033 | FALSE |
| Neighboring Gray Level Dependence - entropy |  |  |  |
| inner vs fringe | 0,006 | 0,017 | TRUE |
| middle vs fringe | 0,033 | 0,033 | FALSE |
| inner vs middle | 0,697 | 0,050 | FALSE |

**S4 Table. *Post-hoc* analysis for the radial configuration for 10 subsets.**  All texture features here reported are the same reported in **Table 4**, and the bold values indicate those texture features that violates the null hypothesis of the *post-hoc* analysis.
