# Supplementary material for: Texture analysis in 177Lu SPECT phantom images: Statistical assessment of uniformity requirements using texture features
Source: PLoS One. 2019 Jul 31;14(7):e0218814. doi: 10.1371/journal.pone.0218814 (PMC6668785; doi:10.1371/journal.pone.0218814)
Supplement: S1 Fig — Reconstructed images of the homogeneous phantom and line profile of pixel counts. A) Central slice of the homogeneous phantom reconstructed with different number of subsets and iterations (reported under each image). B) Counts profile along the dashed yellow line in A) considered for the two datasets. C) Counts profile for 5S1I and 10S1I along the yellow line in A). (DOCX) [file pone.0218814.s007.docx]

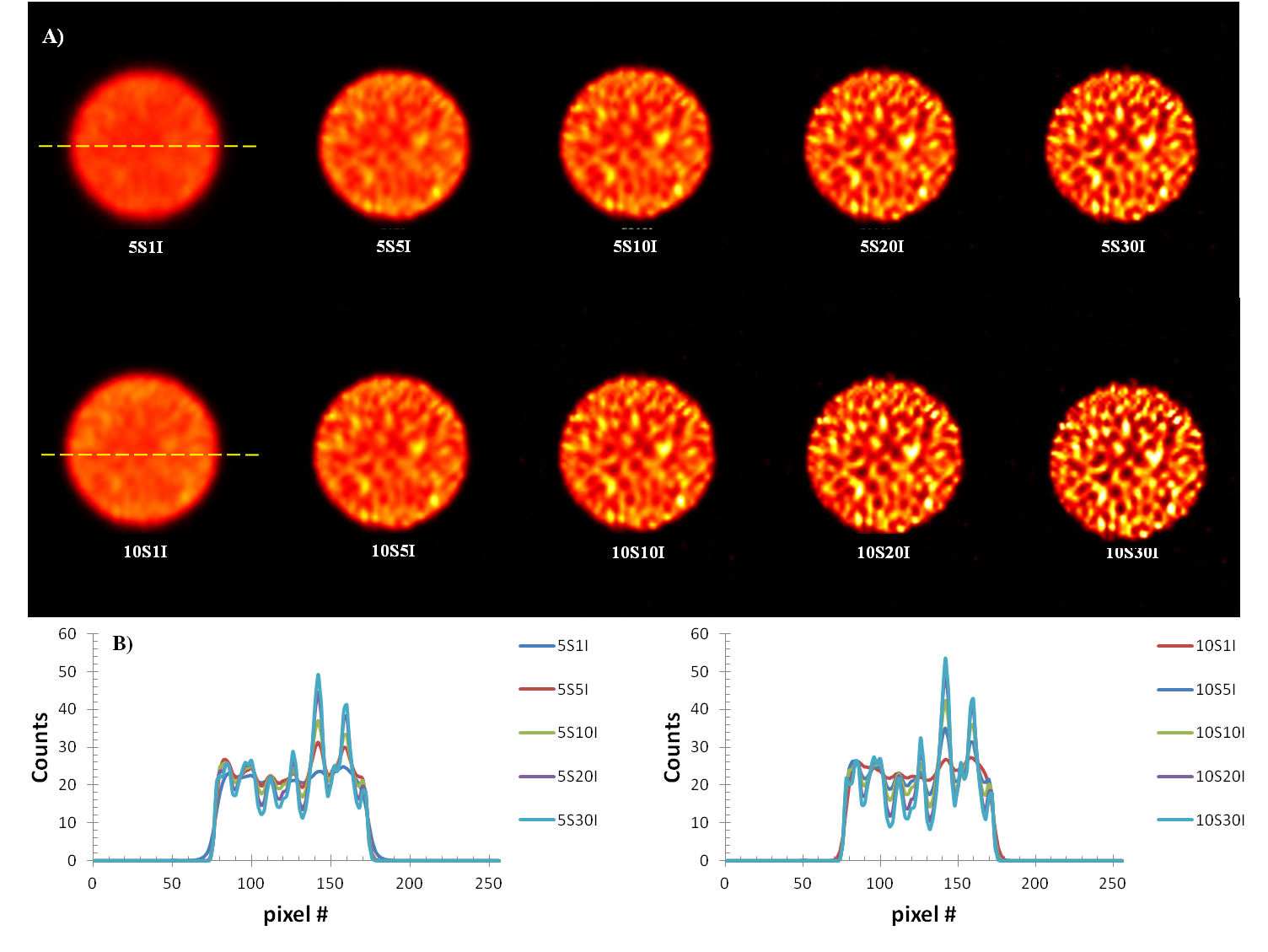


**S1 Figure. Noise profile on reconstructed SPECT images.** Reconstructed images of the homogeneous phantom and line profile of pixel counts. A) Central slice of the homogeneous phantom reconstructed with different number of subsets and iterations (reported under each image). B) Counts profile along the dashed yellow line in A) considered for the two datasets. C) Counts profile for 5S1I and 10S1I along the yellow line in A).


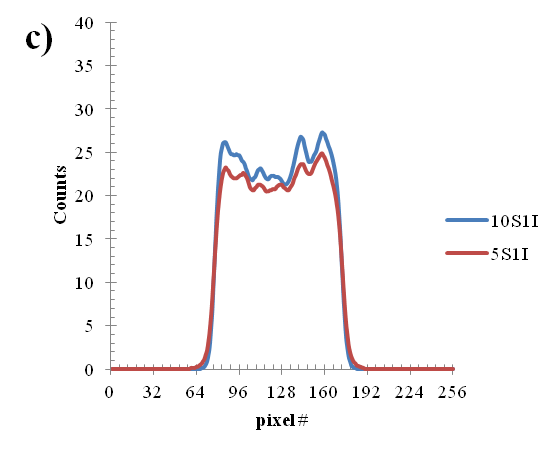


**C)**
